# Biomarker and Drug Target Discovery Using Quantitative Proteomics Post-Intracerebral Hemorrhage Stroke in the Rat Brain

**DOI:** 10.1007/s12031-018-1206-z

**Published:** 2018-11-14

**Authors:** Shuixiang Deng, Shengjie Feng, Wei Wang, Feng Zhao, Ye Gong

**Affiliations:** Department of Intensive Care Unit, HuaShan Hospital, Fudan University, 12 of Wu Lu Mu Qi Middle Road, Shanghai, 200040 China

**Keywords:** Biomarkers, Acute intracerebral hemorrhage, Proteomics, Albumin, ERK

## Abstract

The pathological mechanisms of acute intracerebral hemorrhage (ICH) remain unknown and unverified. In the present study, we used quantitative proteomics to elucidate the pathological mechanisms and to identify novel biomarker and therapeutic target candidates via tissue proteome in a rat model of acute ICH. Rats were experimentally induced with ICH (*n* = 6) or Sham (*n* = 6), and their brain tissue was obtained by 24 h. The TMT-LC–MS/MS-based proteomics approach was used to quantify the differential proteomes across brain tissue, and the results were further analyzed by ingenuity pathway analysis to explore canonical pathways and the relationship involved in the uploaded data. Upon quantification, we found that 96 secreted proteins that were identified in the ICH 24-h group were significantly different those in the control group (*P* < 0.05); among these proteins, 57 increased and 39 decreased in abundance. Bioinformatic analyses of differentially expressed proteins demonstrated that the protein localization and ERK1 and ERK2 cascade were the top two biological processes with the highest concentrations of differentially proteins. The top protein-protein action network with high confidence levels of protein was the albumin and ERK signaling pathways. Albumin, ERK, and p-ERK were assessed in brain tissue by western blot analysis, and higher expression levels of albumin and p-ERK were observed in the ICH group. Our proteomic results highlight important change in the biological processes of ERK1 and ERK2 cascade, which are possible targets for future interventions of ICH. To our knowledge, this study provides in-depth analysis of ICH in brain tissue, and we propose 96 new biomarker candidates for ICH, including albumin and ERK.

**Electronic supplementary material:**

The online version of this article (10.1007/s12031-018-1206-z) contains supplementary material, which is available to authorized users.

## Introduction

Spontaneous intracerebral hemorrhage (ICH) accounts for approximately 10 to 20% of all stroke cases and remains a significant cause of morbidity and mortality throughout the world (Ikram et al. 2012; Qureshi et al. 2001); the 30-day mortality rate is high, functional outcomes are poor, and these values have not substantially changed for decades (Burns et al. 2018; van Asch et al. 2010). Despite its clinical importance, the pathophysiology of ICH is not well understood. Exploring the mechanisms underlying ICH and searching for novel biomarkers and therapeutic targets are important, because ICH leads to poor prognosis and severe psychomotor disability.

To date, no effective surgical or medical treatments have been identified to improve functional outcomes in patients with ICH, largely due to its multiple injury mechanisms (Hemphill et al. 2015; Karsy et al. 2017; Kathirvelu and Carmichael 2015). Numerous preclinical studies have demonstrated that cytotoxicity, excitotoxicity, oxidative stress (OS), and inflammation from the products of red blood cell lysis and plasma components were the main causes of secondary brain injury after ICH, leading to organ damage and brain edema (Duan et al. 2016; Egashira et al. 2015). However, the precise pathophysiological mechanisms underlying ICH remain to be fully elucidated. In recent years, proteomics has emerged as a powerful tool to evaluate the complex host-response to many diseases including ICH (Cao et al. 2014; Li et al. 2017; Martinez-Morillo et al. 2014; Muller et al. 2017; Wang et al. 2017b). This methodology is advantageous for the identification of biomarkers, altered pathways, functional alterations, and mechanisms. Several groups have investigated the proteome changes of cerebrospinal fluid (CSF), plasma and tissue in both ICH patents, and models of ICH (Ren et al. 2014). The timeline of these studies ranged from 3 h to 7 days, but no data on acute 24-h cerebral hemorrhage have been reported. Carmichael compared ICH-induced changes in mRNA expression in perihematomal tissue in rats within 24 h after the hemorrhage. It has been shown that rodent perihematomal tissue networks of pro-inflammatory and anti-inflammatory mediators are activated during the secondary damage period (Lu et al. 2006). However, few studies have examined protein changes in response to ICH-induced brain injury in the early stages, within 24 h, of intracranial infusions of blood into the striatum of adult rats. Therefore, a proteomics profiling of brain tissue 24 h post-ICH will potentially provide insight towards discovering therapeutic target candidates and improving the prognosis of this disease.

We hypothesized that brain tissue substances from an ICH 24-h group would differ from those without ICH and that proteome analysis would identify novel substances associated with ICH. To better understand the molecular pathophysiology of ICH brain injury, this study utilized a TMT-LC-MS/MS-based proteomics approach (Tonack et al. 2009), which is a popular profiling approach in protein biomarker discovery and protein alteration quantification, in order to quantify the differential proteome across brain tissue from ICH. We aimed to disclose possible pathological mechanisms and to identify candidates for novel biomarkers and therapeutic targets involved in ICH via the tissue proteome.

## Materials and Methods

### ICH Models

The experimental design and animal protocols for these studies were approved by the Animal Care Guidelines of the Animal Experimental Committee of Huashan Hospital Fudan University. Male Sprague-Dawley rats weighing 250–300 g were used in this study. Rats were randomly assigned into experimental and control groups. Rat brains were harvested 24 h after ICH. Sham animals went through similar surgical procedures, but ICH was not induced. ICH was produced by the double-injection method described for rats (Alharbi et al. 2016; Deinsberger et al. 1996). The rats were anesthetized with 10% chloral hydrate (0.35-g/kg intraperitoneal injection) and were immobilized in a stereotaxic frame (David Kopf Instruments) 30 min after chloral hydrate injection. A 30-gauge stainless steel cannula was introduced through a burr hole into the right striatum (3 mm lateral to midline, 1.5 mm anterior to bregma, and 6 mm below the surface of the skull), and ICH was induced immediately after cannula was introduced. Each rat received a 10-μL/min^−1^ injection of whole blood (*n* = 6) over 3 min, followed 7 min later by 10-μL/min^−1^ injection over 5 min with a microinfusion pump (KDS-100, kDa Scientific). Fresh autologous blood was taken from the arterial catheter into another polyethylene catheter, the most adequate total hematoma volume turned out to be 100 μl. The injection cannula was slowly withdrawn 10 min after the second injection; the wound was sutured and the animal was placed in an incubator with free access to food and water. Control rats (*n* = 6) had insertion of only the cannula.

Neurological deficits in the rats and magnetic resonance image (MRI) of their skulls were used to assess cerebral hemorrhage after 24 h of intracerebral hemorrhagic surgery. The deficits were scored on a modified system based on the scoring system developed by Longa (Longa et al. 1989) as follows: (0) no deficits, (1) difficulty in fully extending the contralateral forelimb, (2) unable to extend the contralateral forelimb, (3) mild circling to the contralateral side, (4) severe circling, and (5) falling to the contralateral side. Rats that did not display neurologic deficits were removed from the experiment. At 24 h after surgery, the rats were anesthetized with 10% chloral hydrate. Following the anesthetization, the brains were removed and sectioned coronally at the level of the optic chiasm (2 mm posterior to the chiasm) generating one section. In the ICH stroke model, 2-mm-thick slices of rat brain were used for observation of ICH. Only ICH-affected regions were rapidly dissected, rinsed in ice-cold PBS, snap-frozen in liquid nitrogen, and stored at − 80 °C until used.

### Proteomic Analysis

#### Protein Extraction

The sample was resuspended in approximately eight times its volume of lysis buffer (4% SDS, 100-mM Hepes, pH = 7.6, containing phosphatase inhibitor cocktail and PMSF). The homogenate was sonicated for 10 min on ice. After centrifugation at 25,000 *g* for 30 min at 4 °C, the supernatant was collected and stored at − 80 °C. The total protein concentration was measured using a BCA Kit.

#### In-solution Digestion/Labeling

The extracted proteins were mixed according to groups with equal amounts and then precipitated with acetone overnight. After resuspending the proteins in TEAB buffer, protein quantification was performed using the BCA Kit. The proteins samples were reduced by 5-mmol/L DTT at 56 °C for 30 min and alkylated by 10-mmol/L IAA at room temperature for 30 min. Next, the samples were diluted with 50-mmol/L ammonium bicarbonate, until the concentration of urea was lower than 1 M. Trypsin was added to the samples at a mass ratio of 1:50 (enzyme:protein) for 12 h. iTRAQ-8plex labeling reagents (AB Sciex) were added to the peptide samples, which were incubated at room temperature for 120 min. The reaction was stopped with the addition of water, followed by concentration using SpeedVac and desaltings. The purified peptides were collected and stored at − 80 °C until use.

The peptides were fractionated on a water UPLC using a C18 column (Waters BEHC18 2.1 × 50 mm, 1.7 μm). Peptides were eluted at a flow rate of 600 μL/min with a linear gradient of 5–35% solvent B (acetonitrile) over 10 min; solvent A is 20-mM ammonium formate with pH adjusted to 10. The absorbance at 214 nm was determined. A total of 12 fractions were collected and lyophilized.

#### LC-MS/MS Analysis

Each fraction was separated by nano-HPLC (Eksigent Technologies) on a reverse-phase analytical column (Eksigent, C18, 3 μm, 150 mm × 75 μm). Peptides were subsequently eluted using the following gradient conditions with phase B (98% CAN with 0.1% formic acid) from 5 to 45% phase B (5–78 min), and the total flow rate was maintained at 300 nL/min. Electrospray voltage of 2.5 kV versus the inlet of the mass spectrometer was used.

The 5600 mass spectrometer was operated in information-dependent data acquisition mode to switch automatically between MS and MS/MS acquisition. MS spectra were acquired across the mass range of 350–1250 *m*/*z*. The twenty most intense precursors were selected for fragmentation per cycle with a dynamic exclusion time of 30 s.

#### Data Processing

##### Database Searching

All MS/MS samples were analyzed using Mascot (Matrix Science, London, UK; version 2.3.0). Mascot was set up to search the UniProt Human (20,204 entries) database assuming digestion by the enzyme trypsin. Mascot was searched using a fragment ion mass tolerance of 0.1 Da and a parent ion tolerance of 20.0 ppm. Carbamidomethyl of cysteine and iTRAQ8plex of lysine and the N-terminus were specified in Mascot as fixed modifications. Oxidation of methionine and iTRAQ8plex of tyrosine were specified in Mascot as variable modifications.

##### Criteria For Protein Identification

Scaffold (version Scaffold_4.4.5, Proteome Software Inc., Portland, OR) was used to validate MS/MS-based peptide and protein identifications. Peptide identifications were accepted if they achieved an FDR less than 1.0% by the Scaffold Local FDR algorithm. Protein identifications were accepted if they achieved an FDR less than 1.0% and contained at least one identified peptide. Protein probabilities were assigned by the Protein Prophet algorithm (Nesvizhskii et al. 2003). Proteins that contained similar peptides and could not be differentiated based on MS/MS analysis alone were grouped to satisfy the principle.

#### Quantitative Data Analysis

Scaffold Q+ (version Scaffold_4.4.5, Proteome Software Inc., Portland) was used to quantitate iTRAQ peptides and protein identifications. Acquired intensities in the experiment were globally normalized across all acquisition runs. Individual quantitative samples were normalized within each acquisition run. Intensities for each peptide identification were normalized within the assigned protein. The reference channels were normalized to produce a 1:1-fold change. All normalization calculations were performed using medians to multiplicatively normalize data.

#### Bioinformatics

For gene ontology (GO) analysis, we used all of the rat proteins as the basis for calculating enrichment values, which was achieved using a self-written Perl script. A two-tailed Fisher’s exact test and an FDR control were used to test the significance of the enrichment values, which were then represented in a heat map. Pathway analysis was also performed using the Kyoto Encyclopedia of Genes and Genomes (KEGG) pathway database.

#### Ingenuity Pathway Analysis

To further understand the biological significance of differentially expressed plasma proteins, ingenuity pathway analysis (IPA; Ingenuity R Systems, www.Ingenuity.com/) was used to analyze canonical pathways and relationships within the uploaded data. Right-tailed Fisher’s exact test was used to calculate a *P* value to determine the significance of each canonical pathway, and *P* values < 0.05 were considered statistically significant. Disease and functional protein networks and upstream regulator analysis with differentially expressed proteins were presented, along with a *Z* score. A *Z* score ≥ 2 or ≤− 2 was considered significant activation or significant inhibition, respectively.

#### Confirmation of Protein Expression by Western Blot Validation

Validation of LC-MS/MS results of selected proteins in brain homogenates from the sham and ICH rats was performed by means of western blot. Briefly, equal protein amounts (from 50 to 80 μg depending on each specific protein) of brain homogenates were separated on 12.5% polyacrylamide gels (Bio-Rad, Hercules, CA, USA) and then transferred to a PVDF membrane (Millipore). Membranes were blocked for 1 h in 5% skim milk within TBS-T buffer and incubated overnight at 4 °C with the following antibodies: rabbit albumin (1:3000, Abcam, USA), rabbit ERK1/2 (CST1:1000, Abcam, USA), P-ERK1/2 (CST1:2000, Abcam, USA), primary GAPDH antibody (1:3000, Abcam, USA), and the secondary antibody (goat-anti-rabbit IgG conjugated to horseradish peroxidase (1:5000, Abcam, USA)). The specific reaction was visualized by chemiluminescence of the substrate luminal reagent (GE Healthcare, UK). Blot images were then scanned. The optical density of protein levels was quantified using IPP software (version 5.1, Media Lybernetics, USA), corrected for protein load determined by GAPDH, and expressed as the relative value to the controls. Three rats were used in each group.

#### Statistical Analysis

SPSS for Windows, Version 18.0 was used for all statistical analyses. Normally distributed variables are presented as the mean ± standard deviation (SD). Unpaired Student’s *t* test was applied to compare the protein abundance among different groups. Protein abundance of the control group was used as a reference to calculate fold-change; changes of 1.2 or higher and values of *P* values < 0.05 were considered significant.

## Results

### Neurological Deficits and Brain Hemorrhage

**Fig. 1:**
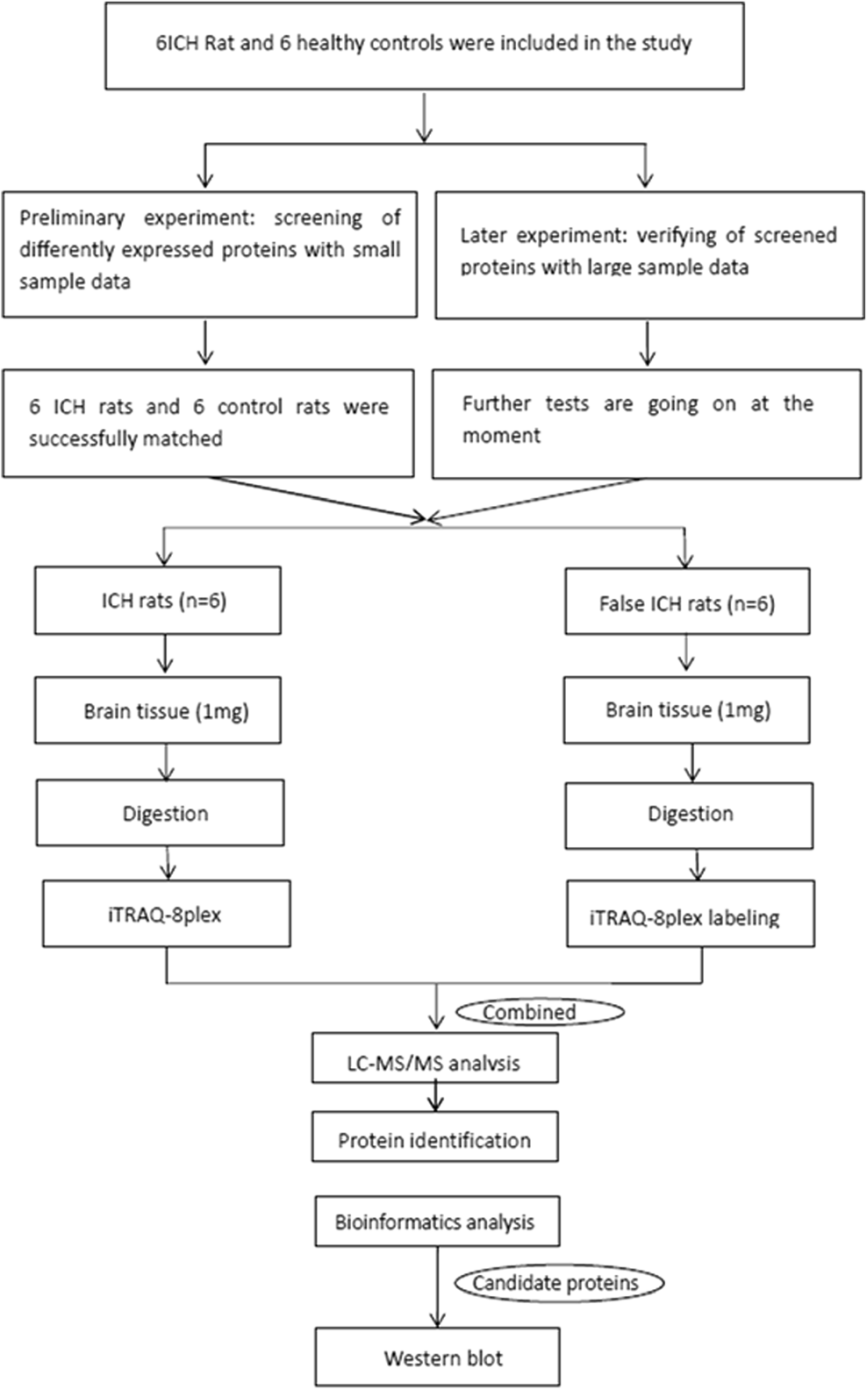
Experimental protocol. The experimental timeline of this study

**Fig. 2:**
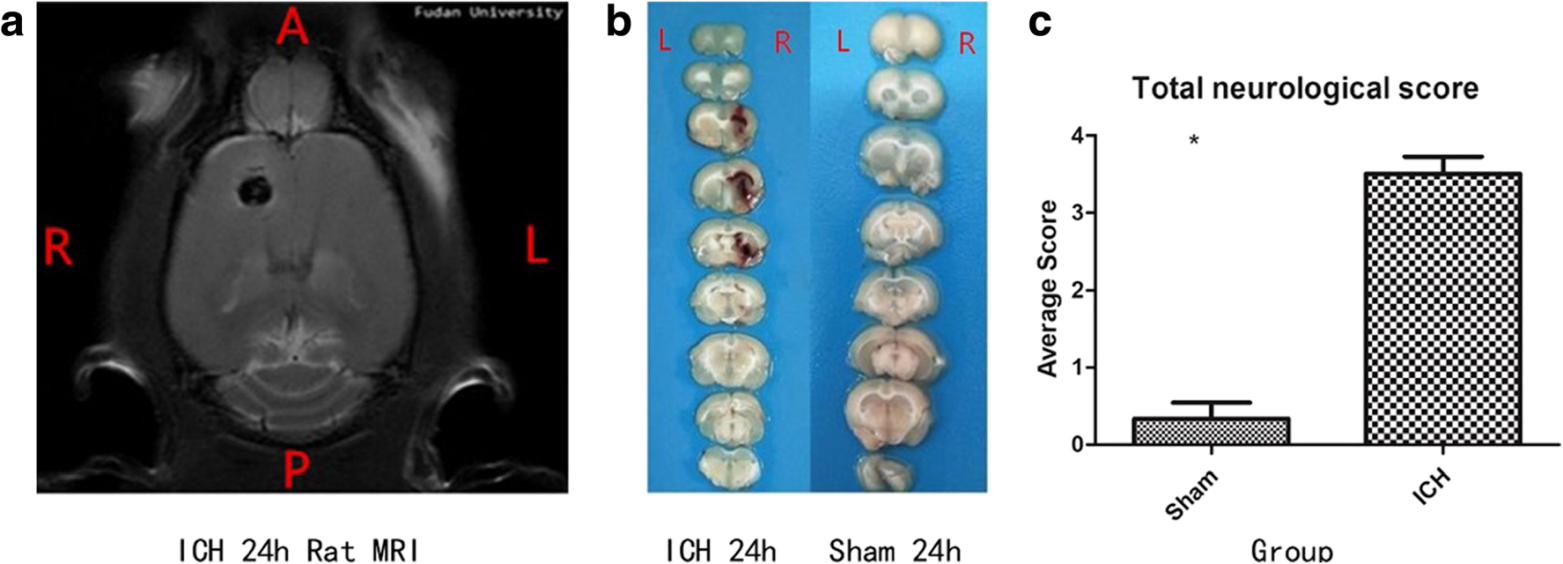
Representative ICH images at 24 h after surgery. Compared with sham rats, experimental rats subjected to ICH 24 h showed clearly visible hematomas in the right striatum. **a** Head MRI showed clearly visible hematomas in the right striatum at 24 h after surgery by a supine anteroposterior position. **b** Pathological section showed clearly visible hematomas in the right striatum at 24 h after surgery. **c** Neurological deficits showed the behavioral score of the experimental ICH group was significantly different from that of the sham group (*P* < 0.05). Total neurological score (normal score = 0; maximal score = 4); *significantly different from sham group (*P* < 0.05); MRI: magnetic resonance image; R: right; L: left; A: anterior; P: posterior

The experimental timeline of this study is shown in Fig. 1. Compared with sham rats, all six ICH-rats showed clearly visible hematomas in the right striatum (Fig. 2). Figure2a head MRI showed clearly visible hematomas in the right striatum after 24 h of intracerebral hemorrhagic surgery by a supine anteroposterior position view; Fig. 2b pathological section showed clearly visible hematomas in the right striatum at 24 h after surgery; and Fig. 2c neurological deficits showed that the behavioral score of the experimental ICH group was significantly different from that of the sham group (*P* < 0.05). The neurological score of the ICH group was 3.5 ± 0.548. The neurological score of the sham group was 0.33 ± 0.516 (Fig. 2).

### Quantification of Brain Tissue Proteins in Sham and ICH Group

**Fig. 3:**
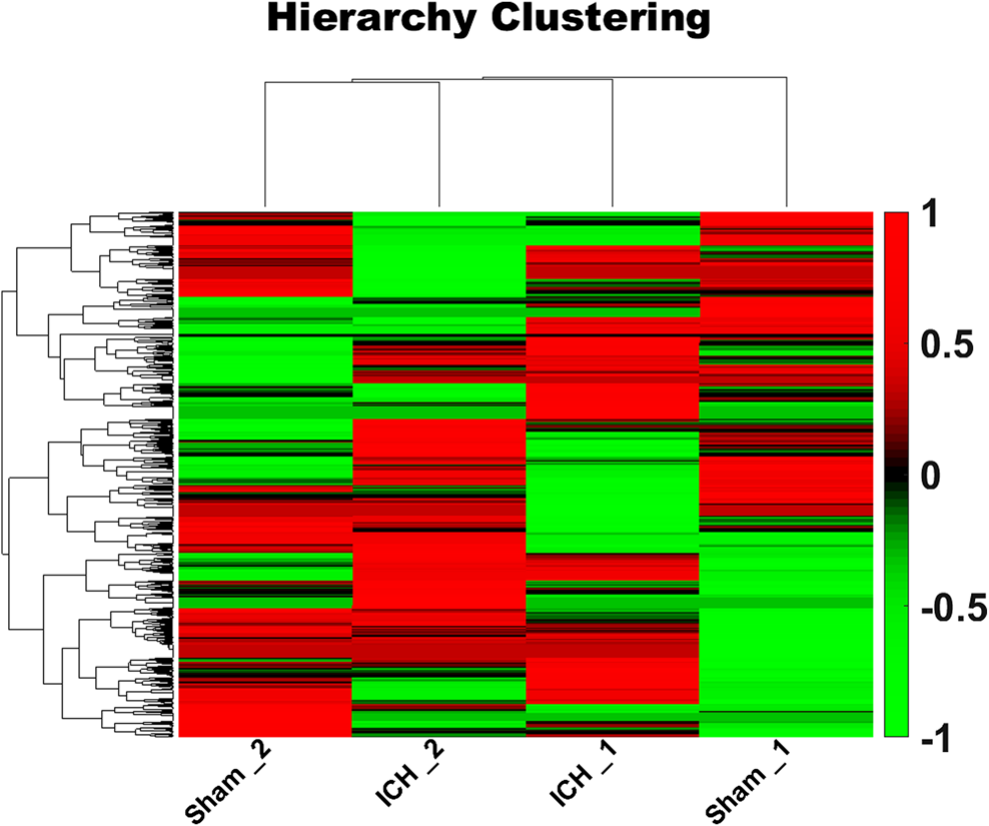
The heat map. Each line is a sample dimension and each row is a genes dimension. The tree on the upper side represents the similarity settlement between samples, and the tree on the left represents the similarity settlement between genes. Red indicates increased, green indicates decreased, and color brightness is directly related to change in level of expression. The heat map shows that a total of 3162 proteins were identified and 96 of the 3162 secreted proteins identified in the ICH 24-h group were significantly differentially expressed from those in the control group (*P* < 0.05); of these proteins, 57 proteins increased while 39 decreased in abundance

For identification of selective proteins secreted in response to experimental ICH, we used the MS-based SILAC approach to quantify the levels of proteins secreted by brain tissue after ICH. A total of 3162 proteins were identified and 96 of 3162 secreted proteins that were identified in the ICH 24-h group were significantly different from those in the control group (*P* < 0.05). Of these proteins, 57 proteins increased while 39 decreased in abundance (Fig. 3). The overall intensity distributions for all proteins are shown in Supplemental supporting information PDF 1. Table 1 lists the ten most highly secreted proteins, and the nine least secreted proteins for which the intensities of the ICH 24-h group were 1.2-fold higher than those of the sham group.

**Table 1:**
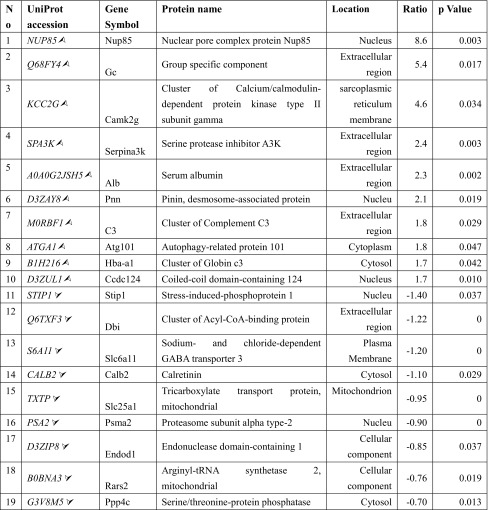
The ten most upregulated proteins and nine most downregulated proteins post-ICH when compared with sham. The table shows proteins that are regulated post-ICH and the level of regulation is expressed as log2 of the iTRAQ ratios

The italicized proteins are identified in our data, and their expression is shown by arrows: upregulation () and downregulation ()

### Bioinformatic Analysis of Secretomics Data

**Fig. 4:**
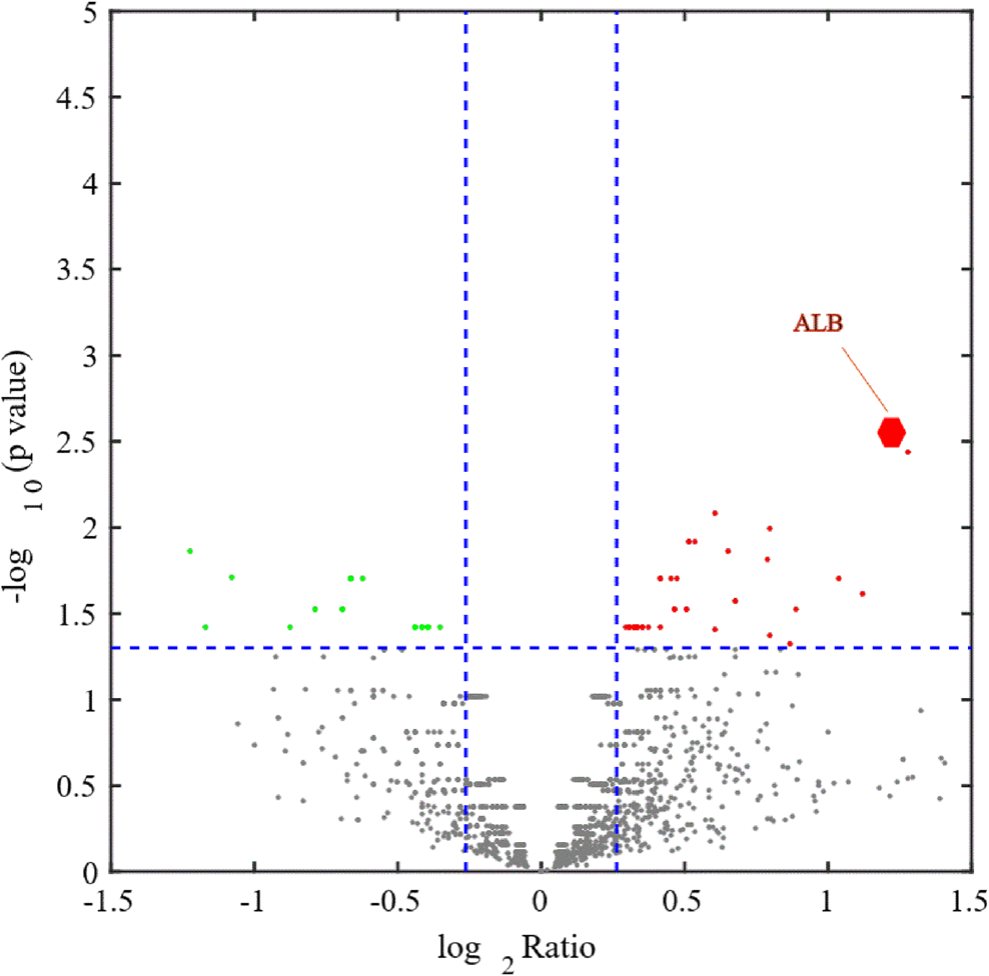
The volcano map. The x-axis represents the protein ratio, the y-axis represents the *P* value of the repeated test results, and each point in the figure represents a protein. The red region contains the upregulation proteins, and the green region contains the downregulation proteins. The volcano map shows the 96 secreted proteins that were identified in the ICH 24-h group; of these proteins, 57 proteins increased while 39 decreased in abundance

**Fig. 5:**
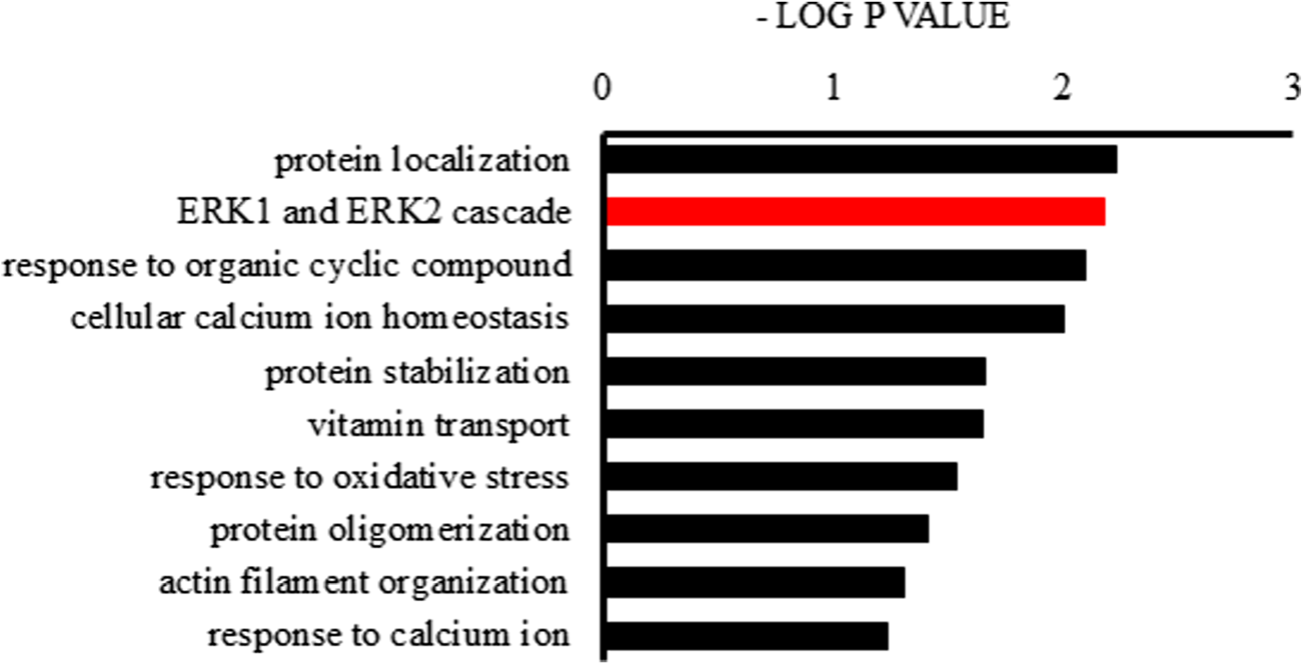
Bioinformatic analysis showed ten biological process items with the highest concentration of differential protein. The top three biological processes with the highest concentration of differentially expressed proteins were protein localization, the ERK1 and ERK2 cascade, and the response to organic cyclic compounds

**Fig. 6:**
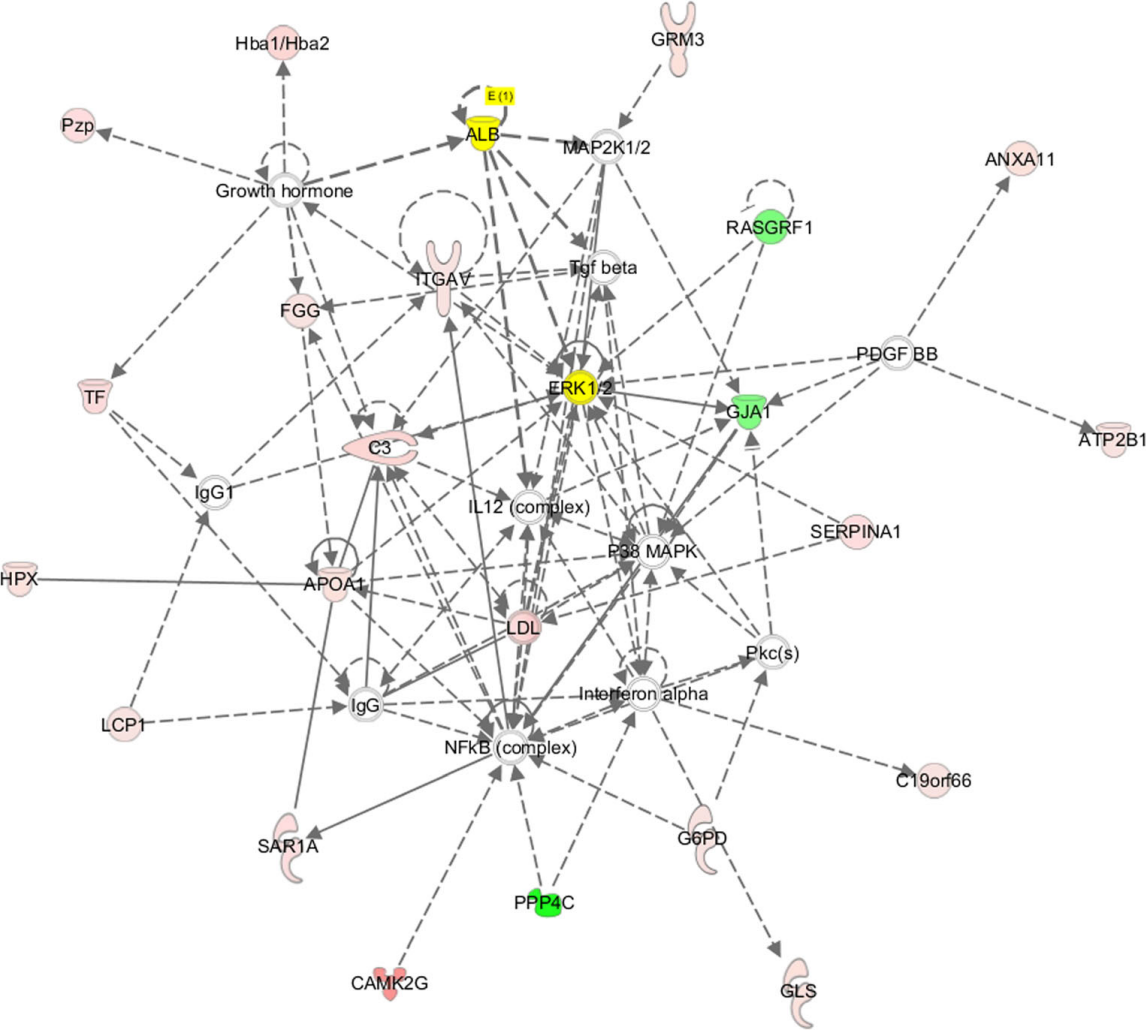
The action networks map. The top action networks with high confidence levels for protein involved in the function of “cardiovascular diseases, organismal injury and abnormalities, and outraged morphology” were albumin and ERK signaling pathways. In these figures, proteins are shown in four colors: red or pink for upregulated and green for downregulated proteins, yellow for highlighting the upregulated albumin and ERK proteins, and white representing the unaffected proteins. Proteins are shown in different shapes based on function. Pathways to which the proteins are related are shown in gray boxes

For each identified protein, we performed GO enrichment analysis. The results are presented in a heat map in Fig. 4. Bioinformatic analysis of differentially expressed proteins showed that the top three biological processes with the highest concentration of differentially expressed proteins were protein localization, the ERK1 and ERK2 cascade, and the response to organic cyclic compounds (Fig. 5). IPA was used to analyze canonical pathways and protein-protein relationships. The top action networks of proteins with high confidence levels were albumin and ERK signaling pathways (Fig. 6 and Table 2). Upstream regulator analysis found two regulators, IL6 and HNF4A, with an activation *Z* score of 2 (Table 3). The roles of the proteins upregulated in brain tissue by ICH were further characterized by KEGG pathway analysis (Supplemental supporting information Fig. 1). The majority of these proteins were associated with Parkinson’s disease, aminoacyl-tRNA biosynthesis and complement and coagulation cascades, and a calcium signaling pathway.

**Table 2:** Major networks and associated proteins obtained by IPA analysis of differentially expressed ICH proteins

Top diseases and functions	Molecules in network	Score focus	Molecules
Cardiovascular disease, organismal injury and abnormalities, organ morphology	↑ALB, ↑ANXA11, ↑ APOA1, ↑ATP2B1, ↑C3, ↑C19orf66, ↑CAMK2G, ERK1/2, ↑FGG, ↑G6PD, ↓GJA1, ↑GLS, ↑GRM3, growth hormone, ↑Hba1/Hba2, ↑HPX, IgG, IgG1, IL12 (complex), interferon alpha, ↑ITGAV, ↑LCP1, LDL, MAP2K1/2, NFkB (complex), P38MAPK, PDGFBB, Pkc(s), ↓PPP4C, ↑Pzp, ↓RASGRF1, ↑SAR1A, ↑SERPINA1, ↑TF, Tgf beta	43	22

The italicized proteins are identified in our data, and their expression is shown by arrows: upregulation (↑) and downregulation (↓)

**Table 3:** Upstream regulator and associated proteins obtained by IPA analysis of differentially expressed ICH proteins

Upstream regulator	Molecule type	Activation *Z* score	*P* value	Target molecules in dataset
IL6	Cytokine	1.436	0.00187	ALB, C3, FGG, HPX, SERPINA1, TF
HNF4A	Transcription regulator	1.4	0.0156	APOA1, HPX, PEPD, SERPINA1, TF

### Confirmation of Secreted Albumin and ERK

**Fig. 7:**
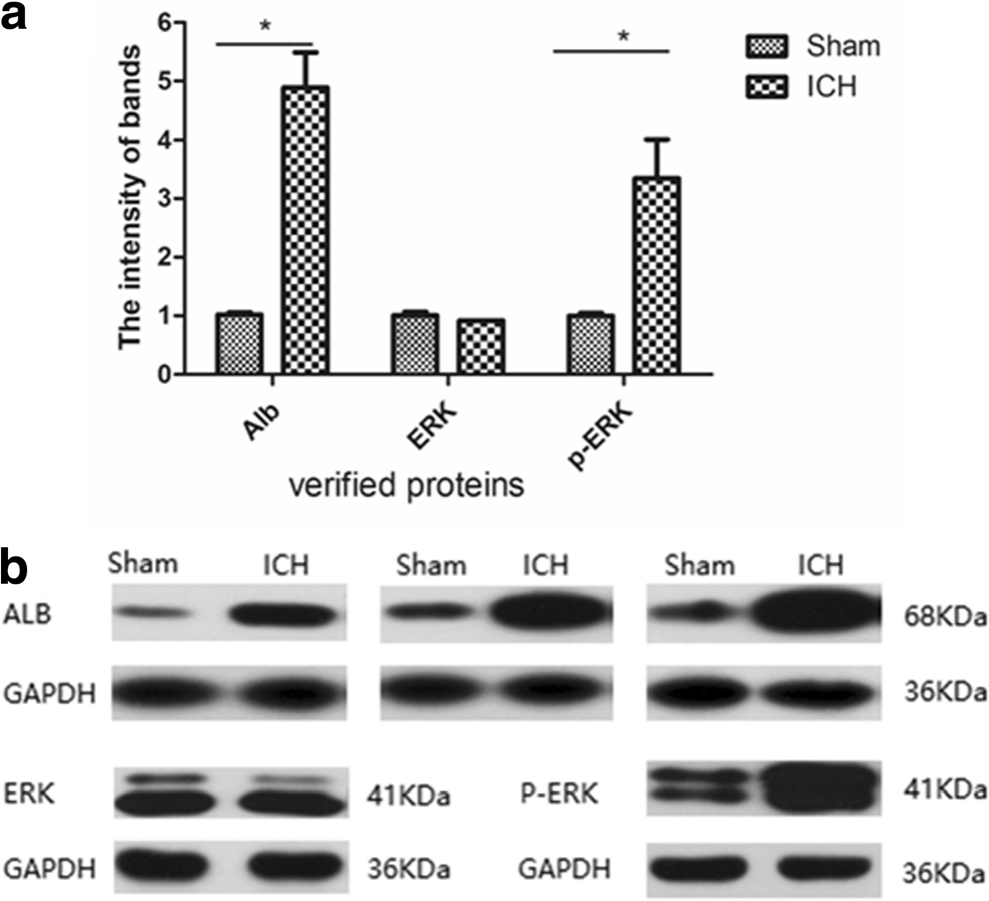
The western blot result of albumin and ERK. The western blot shows higher expression levels of albumin and p-ERK observed in the ICH group compared to the sham group (*P* < 0.05), but there was no difference of ERK between ICH group and sham group (*P* > 0.05). GAPDH is the internal standard reference group. **a** The intensity of each band was measured using imaging analysis.*P < 0.05 versus control. **b** Western blotting analysis of the three proteins

Western blot validation was conducted to further confirm protein identification and quantification in ICH rats. According to the data from iTRAQ, out of the 57 highly secreted proteins, we focused on the secretion profile of albumin, which is a unique pleiotropic protein with multiple properties, and ERK. The western blot results in each group are shown in Fig. 7, and higher expression levels of albumin and p-ERK were observed in the ICH group compared to the sham group (*P* < 0.05).

## Discussion

Experimental ICH is commonly induced in rodents through the injection of autologous blood, which can produce consistent neurological deficit, hypoperfusion, hematoma volume, and brain swelling. This model closely mimics human hypertensive basal ganglionic ICH and can be useful for evaluating the efficacy of pharmaceutical therapies (Belayev et al. 2003; Deinsberger et al. 1996). To the best of our knowledge, this is the first comprehensive study to analyze brain tissue 24 h following a model of ICH induction with autologous blood. Here, we used high-throughput protein identification and quantification by LC-MS/MS proteomics to identify ICH biomarkers. Brain tissue analysis revealed that the expression of 96 proteins was significantly different between ICH rats and sham rats during the early stage of ICH; of those proteins, 57 were upregulated, accounting for approximately half of the protein changes. The identification, classification and analysis of protein groups could shed light on the molecular basis of injury prognosis after ICH. Here, we propose 96 new biomarker candidates for ICH, including albumin and ERK.

ICH is associated with high mortality and morbidity, and the underlying mechanisms are complex, consisting of cytotoxic, excitotoxic, and inflammatory effects of in traparenchymal blood, and are responsible for its highly damaging effects (Hu et al. 2016). OS appears to play a prominent role in ICH pathogenesis (Aronowski and Zhao 2011). Increasing evidence demonstrates that the metabolite axis of hemoglobin-heme-iron is the key contributor to oxidative brain damage after ICH, although other factors, such as neuroinflammation and peroxidases, are involved (Duan et al. 2016). Two quantitative proteomics studies of ICH demonstrated that proteins involved in OS were upregulated with in ICH, while endogenous antioxidants were downregulated (Martinez-Morillo et al. 2014). These proteomics may be potential therapeutic targets for ICH.

Albumin, a unique pleiotropic protein with multiple properties is known for its transportation of a wide range of endogenous ligands and drugs, and its actions as a potent antioxidant with free radical scavenging activities due to its unique biochemical structure (Anraku 2014; Sitar et al. 2013). Albumin has been used to treat many clinical conditions, but the specific mechanism remains unclear. Hypoalbuminemia is a frequent finding in acute ischemic stroke and has been associated with increased stroke severity and poor clinical outcomes (Limaye et al. 2016; Morotti et al. 2017; Tsao et al. 2011; Wang et al. 2017a). One reason that hypoalbuminemia after stroke may reduce the synthesis of protein post-stroke is due to poor diet. Another reason may be that the vascular permeability increase that leads to the loss of albumin increases after stroke due to the complex cascade of secondary brain damage, such as inflammatory injury, OS, and cytotoxic damage. Although the precise mechanisms have not been fully described, serum albumin has protective effects such as antioxidant activity, maintenance of physiologic homeostasis, and anti-inflammatory effects. Therefore, these protective biological functions may be impaired in conditions of hypoalbuminaemia, and therefore, increased morbidity and mortality can consequently develop in stroke patients.

In our study, out of the 57 highly secreted proteins, we focused on the secretion profile of albumin which is a unique pleiotropic protein with multiple properties according to the data from iTRAQ. The western blot results showed a higher expression of albumin in the ICH group. Ingenuity pathway analysis showed that the top networks with high confidence levels include albumin and ERK signaling pathways involved in cardiovascular disease, organismal injury and abnormalities, and organ morphology. Further characterization by KEGG pathway analysis showed the majority of these upregulated proteins were associated with a calcium signaling pathway. Ahn, Sung-Min and his colleagues found evidence in a pilot phase study of the Human Brain Proteome Project that microglial cells in the brain may synthesize albumin and these cells may play a beneficial role in Alzheimer’s disease by secreting albumin (Ahn et al. 2008). Therefore, the potential mechanisms underlying elevated albumin presence in the brain may be mainly attributed to its local release rather than its crossing of the blood-brain barrier. These results suggest that albumin plays an important role in cerebral hemorrhage and may be involved in antioxidant effects. Albumin can be used as a biomarker and therapeutic target for cerebral hemorrhage.

IPA created an interaction network among the differentially expressed proteins, and signaling proteins revealed interactions between their functions and the disease. In this study, bioinformatic analyses showed that the top biological processes with the highest concentration of differentially expressed proteins were ERK1 and ERK2 cascades. IPA showed that the top action networks of proteins with high confidence levels were ERK signaling pathways. The western blot results showed a higher expression of p-ERK. Amazingly, no ICH-related networks have been confirmed based on the existing IPA knowledge database, illustrating that the identified proteins have not been previously reported in a network with functions related to ICH. Additionally, the mitogen-activated protein kinases/extracellular signal-regulated kinase (MAPK/ERK) pathway is suggested to be a key regulator in the network, as it regulated the majority (81/97) of the proteins in this study. The MAPK/ERK pathway is reported to be associated with cell proliferation, differentiation, migration, senescence, and apoptosis. P38MAPKs, one member of the MAPK family, is responsible for signal transmission between extracellular space and the nucleus, leading to biological cell reactions (Sun et al. 2015). A number of studies demonstrate that p38MAPK signaling is an important inflammatory pathway in subarachnoid hemorrhage (SAH) through various mechanisms (Muller et al. 2017). Zhao et al. found that p38MAPK was activated at 24 h in an animal model of ICH, and the use of an inhibitor could recede the inflammatory injury after ICH (Zhou et al. 2014). Boehme AK et al. showed systemic inflammatory response syndrome (SIRS) on admission was associated with an ICH score on admission and infection after ICH infection (Boehme et al. 2018). Together, MAPK/ERK and p38MAPK could represent potential therapeutic targets to prevent neurological deficits after ICH.

This study has several limitations. Although we identified and validated two novel substances of ICH, the present study is a pilot study of a comprehensive proteome analysis; thus, the sample size was small, similar to that of previous proteome studies (Ren et al. 2014). We have collected more than 200 serum samples from ICH patients in a hospital to conduct further research. In this study, we discussed only the tissue proteomic changes in rats 24 h after ICH.

## Conclusion

In summary, we identified 96 proteins that were highly secreted in brain tissue in response to ICH, and we used western blot to confirm the specific secretion of albumin and ERK in response to ICH. Our proteomic results stress that important changes occur in the biological processes of protein localization, the ERK1 and ERK2 cascade, and the response to organic cyclic compounds, which are possible targets for future interventions of ICH. To our knowledge, this study provides the first in-depth analysis of brain tissue from 24 h post-ICH, and we propose 96 new biomarker candidates for ICH, including albumin and ERK.

## Electronic Supplementary Material

ESM 1 (GIF 9 kb)

ESM 2 (PDF 224 kb)
