# Supplementary figures and images for: Biomarker and Drug Target Discovery Using Quantitative Proteomics Post-Intracerebral Hemorrhage Stroke in the Rat Brain
Source: J Mol Neurosci. 2018 Nov 14;66(4):639–48. doi: 10.1007/s12031-018-1206-z (PMC6267379; doi:10.1007/s12031-018-1206-z)

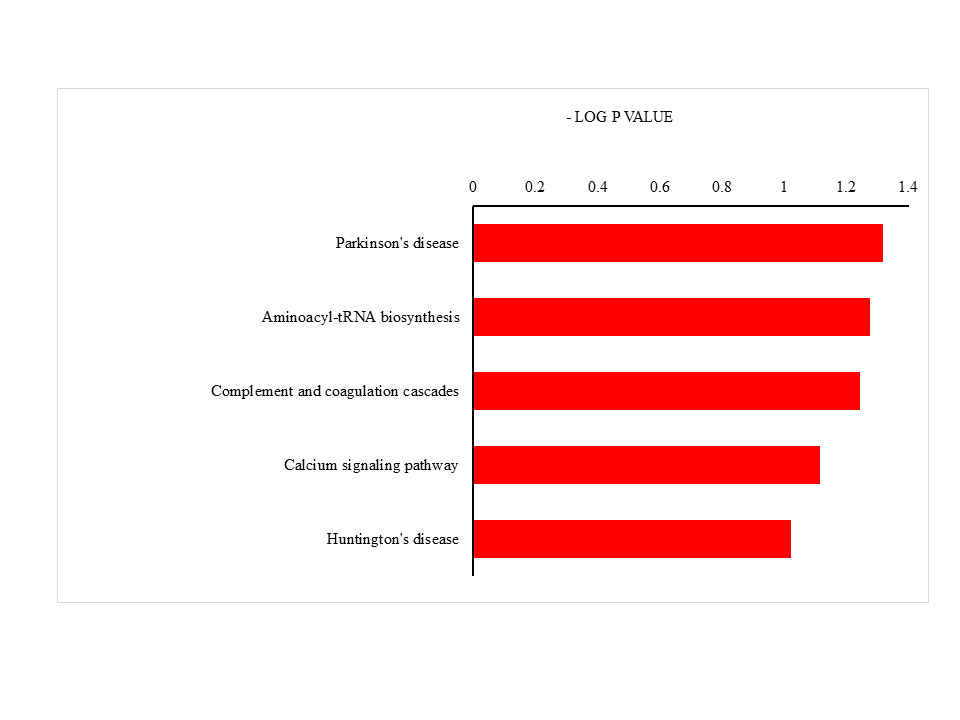

Supplement: Supplementary file 1 — (GIF 9 kb) [file 12031_2018_1206_MOESM1_ESM.gif]
