# Supplementary material for: Biomarker and Drug Target Discovery Using Quantitative Proteomics Post-Intracerebral Hemorrhage Stroke in the Rat Brain
Source: J Mol Neurosci. 2018 Nov 14;66(4):639–48. doi: 10.1007/s12031-018-1206-z (PMC6267379; doi:10.1007/s12031-018-1206-z)
Supplement: Supplementary file 2 — (PDF 224 kb) [file 12031_2018_1206_MOESM2_ESM.pdf]

INGENUITY PATHWAY ANALYSIS

Analysis Name: DSX_AB - 2018-02-05 07:29
Analysis Creation Date: 2018-02-05
Build version: 463341M
Content version: 42012434 (Release Date: 2017-12-07)

Analysis Settings

Reference set: Ingenuity Knowledge Base (Genes Only)

Relationship to include: Direct and Indirect

Does not Include Endogenous Chemicals

Optional Analyses:

Filter Summary:

Consider only molecules and/or relationships where

(species = Human) AND

(confidence = Experimentally Observed)

(c) 2000-2018 QIAGEN. All rights reserved.

Top Canonical Pathways

Name	p-value	Overlap
LXR/RXR Activation	3.36E-08	6.6 % 8/121
FXR/RXR Activation	8.27E-07	5.6 % 7/126
Acute Phase Response Signaling	6.09E-06	4.1 % 7/170
Clathrin-mediated Endocytosis Signaling	1.63E-04	3.0 % 6/199
tRNA Charging	5.35E-04	7.7 % 3/39

Top Upstream Regulators

Upstream Regulator	p-value of overlap	Predicted Activation
Growth hormone	6.98E-05	
SREBF1	1.69E-04	
PPARA	2.37E-04	
HTT	6.37E-04	
PAX2	1.44E-03	

Top Diseases and Bio Functions

Diseases and Disorders

Name	p-value	#Molecules
Cardiovascular Disease	1.62E-02 - 4.81E-06	20
Organismal Injury and Abnormalities	1.62E-02 - 4.81E-06	53
Cancer	1.10E-02 - 1.24E-05	37
Neurological Disease	1.62E-02 - 1.24E-05	44
Gastrointestinal Disease	1.22E-02 - 5.26E-05	22

Molecular and Cellular Functions

(c) 2000-2018 QIAGEN. All rights reserved.

INGENUITY PATHWAY ANALYSIS

Name	p-value	#Molecules
Cell-To-Cell Signaling and Interaction	1.61E-02 - 8.20E-07	26
Lipid Metabolism	1.59E-02 - 4.94E-06	18
Small Molecule Biochemistry	1.59E-02 - 4.94E-06	25
Vitamin and Mineral Metabolism	1.22E-02 - 4.94E-06	14
Molecular Transport	1.59E-02 - 1.81E-05	37

Physiological System Development and Function

Name	p-value	#Molecules
Nervous System Development and Function	1.61E-02 - 8.20E-07	18
Renal and Urological System Development and Function	4.07E-03 - 2.67E-05	6
Hair and Skin Development and Function	1.22E-02 - 3.66E-05	8
Organ Morphology	1.62E-02 - 5.08E-05	16
Organismal Development	1.62E-02 - 5.08E-05	29

Top Tox Functions

Assays: Clinical Chemistry and Hematology

Name	p-value	#Molecules
Decreased Levels of Albumin	4.77E-02 - 7.49E-03	2

Cardiotoxicity

Name	p-value	#Molecules
Congenital Heart Anomaly	2.14E-01 - 1.17E-03	6
Cardiac Arrythmia	3.04E-01 - 4.07E-03	2
Cardiac Proliferation	2.73E-01 - 4.07E-03	1
Cardiac Infarction	2.11E-01 - 4.27E-03	5
Cardiac Inflammation	1.22E-02 - 1.22E-02	1

(c) 2000-2018 QIAGEN. All rights reserved.

INGENUITY PATHWAY ANALYSIS

Hepatotoxicity

Name	p-value	#Molecules
Liver Cholestasis	2.14E-01 - 5.26E-05	5
Liver Cirrhosis	1.67E-02 - 1.05E-03	4
Liver Damage	1.75E-01 - 4.07E-03	4
Liver Hyperplasia/Hyperproliferation	1.00E00 - 4.07E-03	47
Liver Fibrosis	1.37E-01 - 6.18E-03	4

Nephrotoxicity

Name	p-value	#Molecules
Renal Inflammation	5.11E-01 - 2.17E-03	3
Renal Nephritis	5.11E-01 - 2.17E-03	3
Renal Damage	1.33E-01 - 6.48E-03	5
Renal Tubule Injury	6.48E-03 - 6.48E-03	3
Renal Dilation	3.99E-02 - 2.02E-02	1

Top Networks

ID	Associated Network Functions	Score
1	Cardiovascular Disease, Organismal Injury and Abnormalities, Organ Morphology	43
2	Cellular Development, Cardiovascular System Development and Function, Organismal Development	26
3	Hair and Skin Development and Function, Organ Development, Lipid Metabolism	24
4	Cellular Assembly and Organization, Gene Expression, Infectious Diseases	21
5	Cellular Function and Maintenance, Drug Metabolism, Lipid Metabolism	21

(c) 2000-2018 QIAGEN. All rights reserved.

INGENUITY PATHWAY ANALYSIS

Top Tox Lists

Name	p-value	Overlap	
LXR/RXR Activation	3.82E-08	6.5 %	8/123
FXR/RXR Activation	8.27E-07	5.6 %	7/126
Negative Acute Phase Response Proteins	3.59E-06	37.5 %	3/8
Positive Acute Phase Response Proteins	6.46E-06	13.3 %	4/30
Increases Liver Steatosis	7.92E-03	3.0 %	3/100

Top Analysis-Ready Molecules

Expr Fold Change up-regulated

Molecules	Expr. Value	Expr. Chart
**NUP85**	↑ 8.600	
**GC**	↑ 5.412	
**CAMK2G**	↑ 4.600	
**Serpina3c/Serpina3m**	↑ 2.423	
**ALB**	↑ 2.368	
**PNN**	↑ 2.050	
**C3**	↑ 1.850	
**ATG101**	↑ 1.824	
**Hba1/Hba2**	↑ 1.739	
**CCDC124**	↑ 1.737	

Expr Fold Change down-regulated

Molecules	Expr. Value	Expr. Chart
**PPP4C**	↓ -2.333	
**LARS2**	↓ -2.250	
**RARS2**	↓ -2.111	
**FAM98B**	↓ -1.833	
**SNAP29**	↓ -1.727	

(c) 2000-2018 QIAGEN. All rights reserved.

INGENUITY PATHWAY ANALYSIS

**RAB27B**	↓ -1.727
**SYAP1**	↓ -1.615
**HNRNPA0**	↓ -1.615
**CALB2**	↓ -1.615
**SNX17**	↓ -1.583

(c) 2000-2018 QIAGEN. All rights reserved.

INGENUITY PATHWAY ANALYSIS
